# Supplementary figures and images for: Sequencing mRNA from Cryo-Sliced Drosophila Embryos to Determine Genome-Wide Spatial Patterns of Gene Expression
Source: PLoS One. 2013 Aug 12;8(8):e71820. doi: 10.1371/journal.pone.0071820 (PMC3741199; doi:10.1371/journal.pone.0071820)

## 60 $\mu$ m Replicate 1

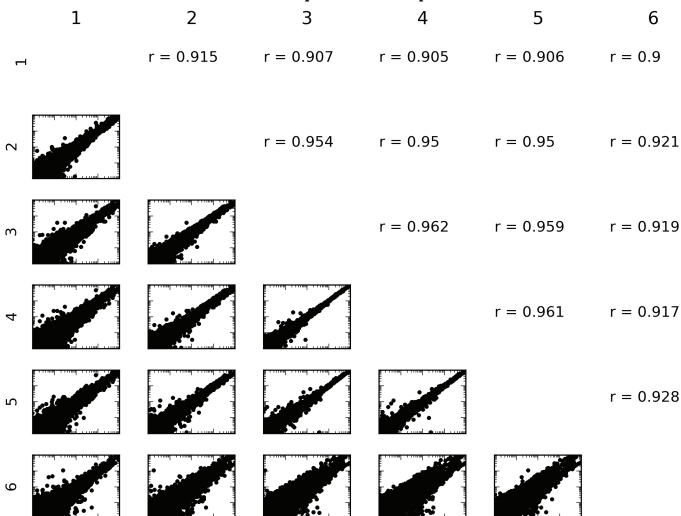

## 60 $\mu$ m Replicate 2

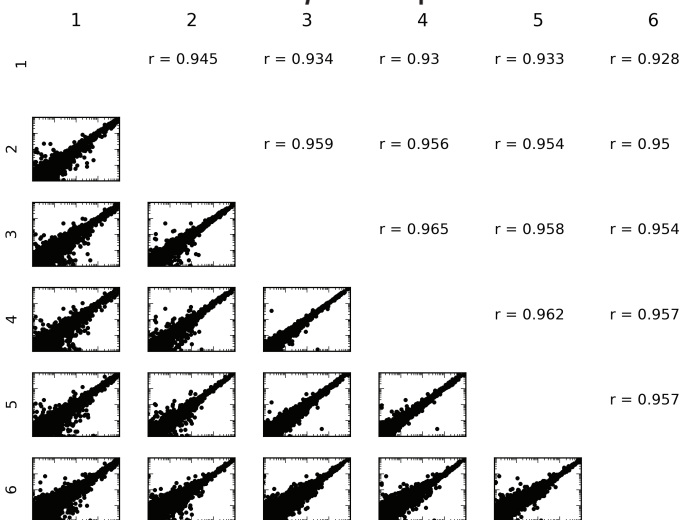

## 60 $\mu$ m Replicate 3

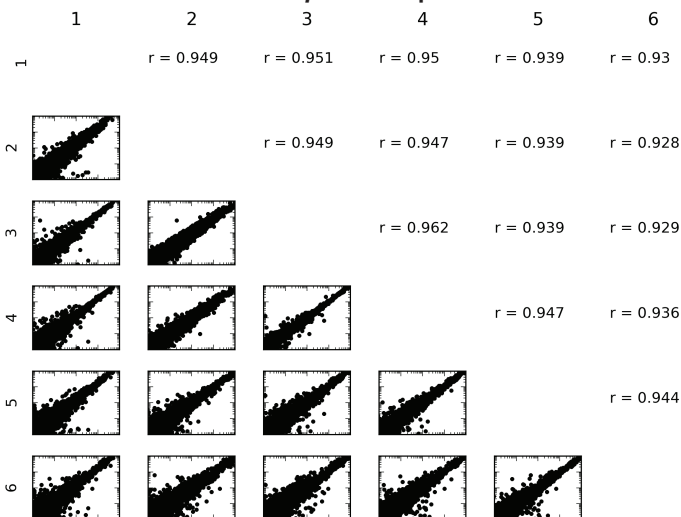

Supplement: Figure S1 — Correlation of slices within embryos. Log-log plots of FPKM values between slices within each of the three 60 µm sliced embryos. (PDF) [file pone.0071820.s001.pdf]

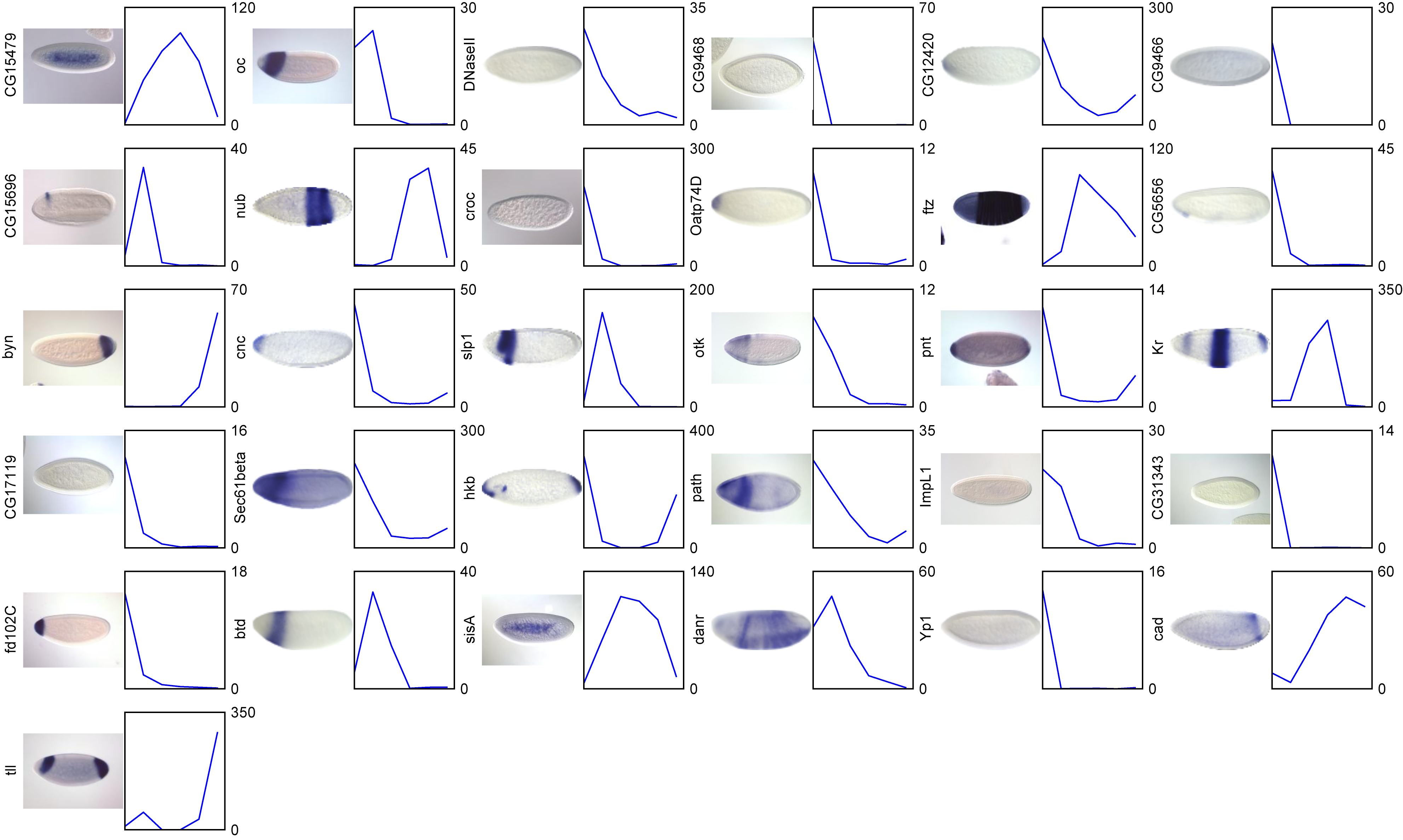

Supplement: Figure S3 — Genes called as patterned by Cuffdiff lacking subset tag in BDGP database. Images are from BDGP; graphs are average of three CaS embryos. Many of these are known patterned genes, highlighting the incompleteness of available annotations. (TIF) [file pone.0071820.s003.tif]

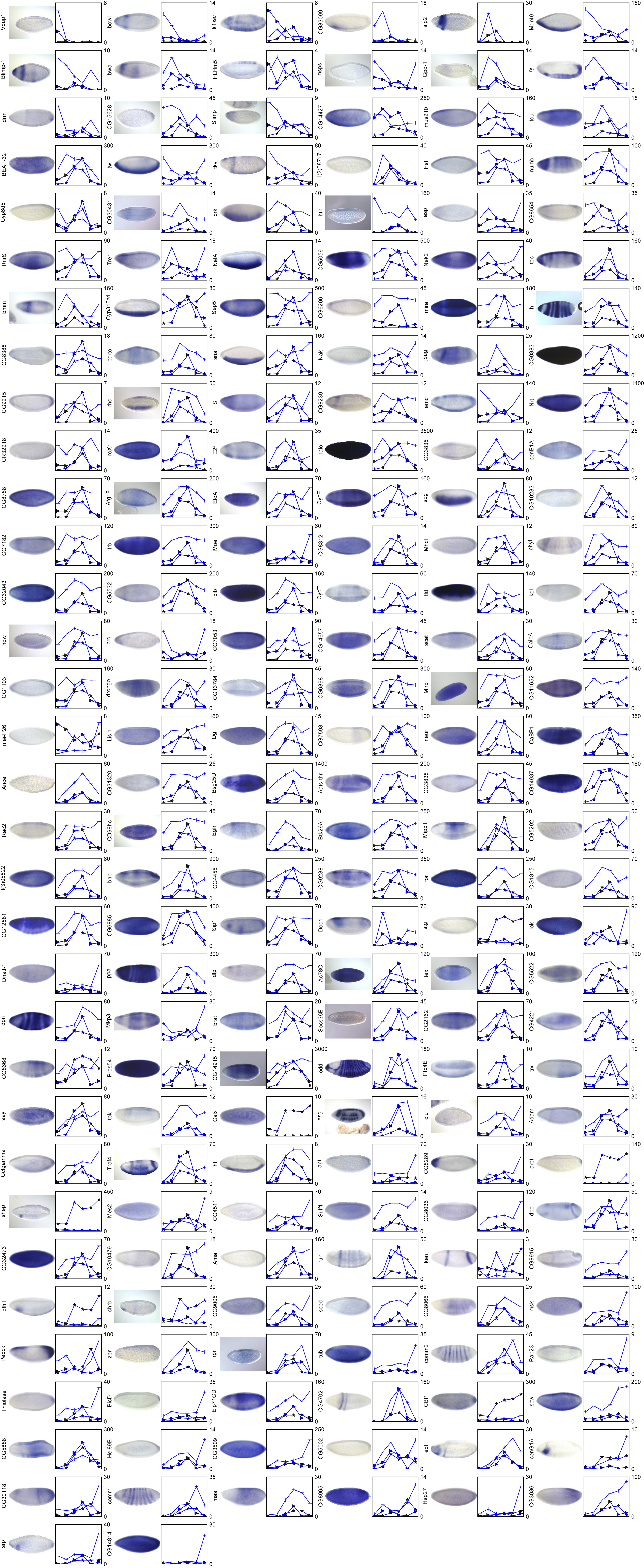

Supplement: Figure S4 — Genes with subset tag in BDGP not called as patterned by Cuffdiff. (TIF) [file pone.0071820.s004.tif]

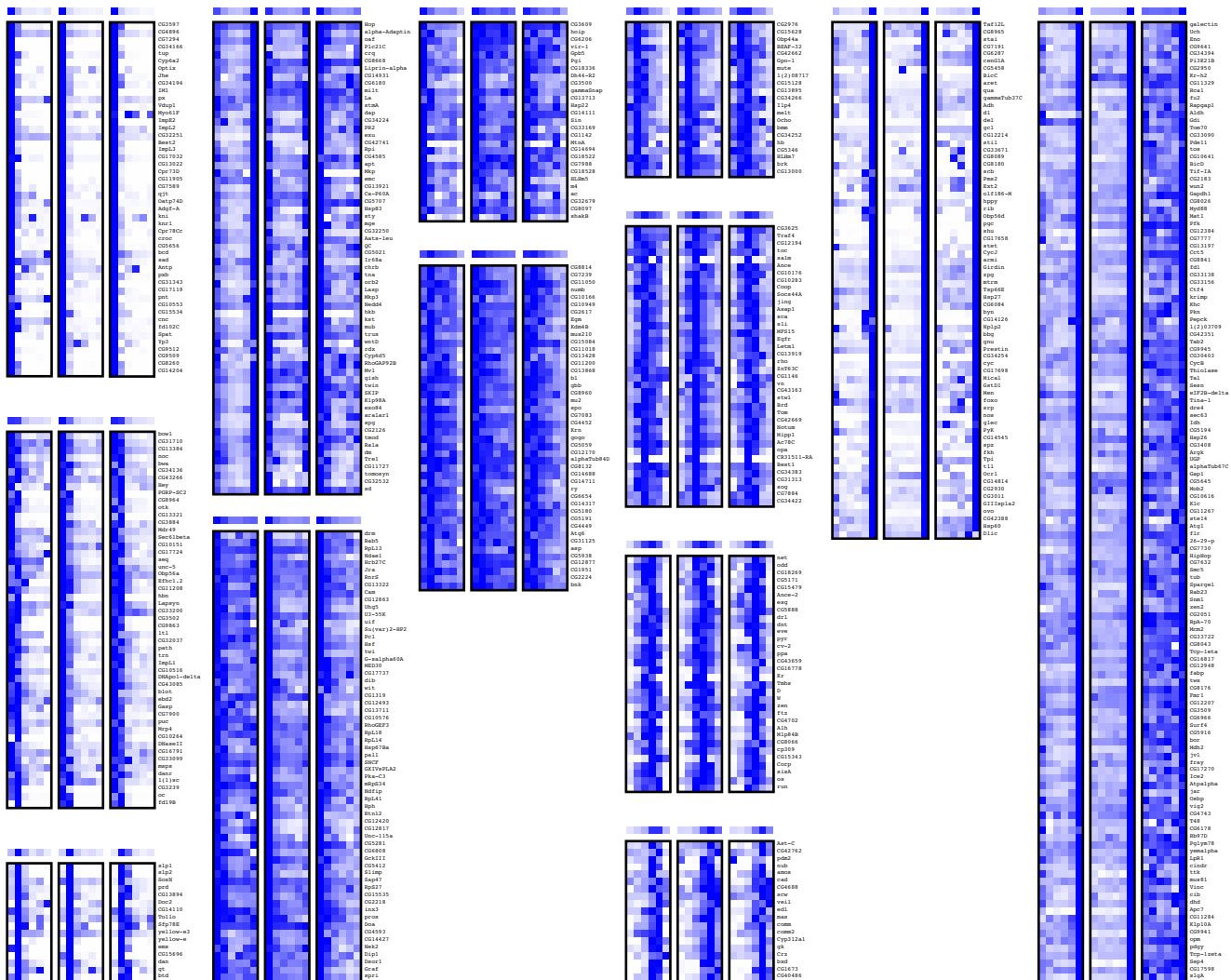

Supplement: Figure S5 — Figure 2 with gene names. (PDF) [file pone.0071820.s005.pdf]

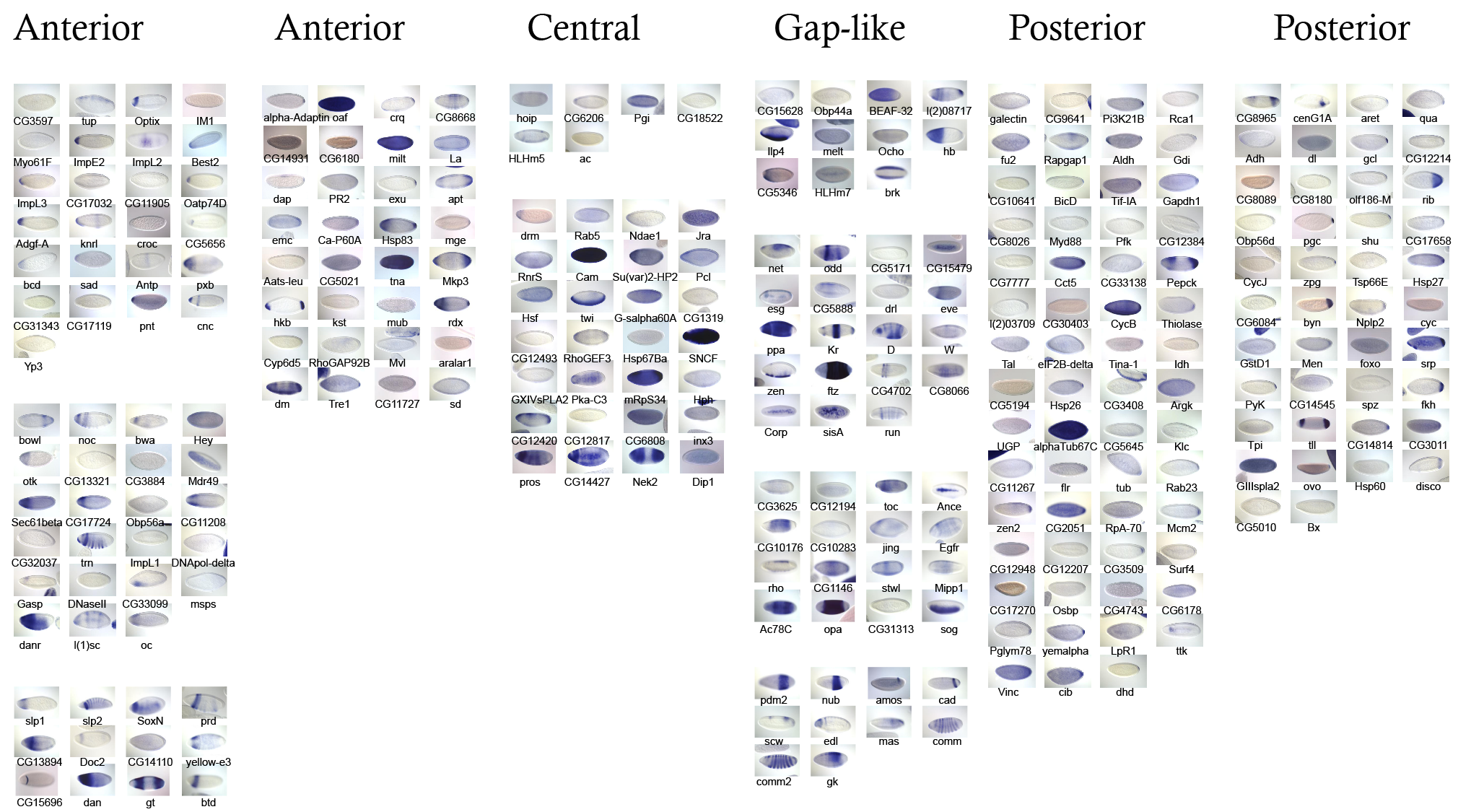

Supplement: Figure S6 — Images from BDGP for genes in clusters shows in Figure 2 . (TIF) [file pone.0071820.s006.tif]

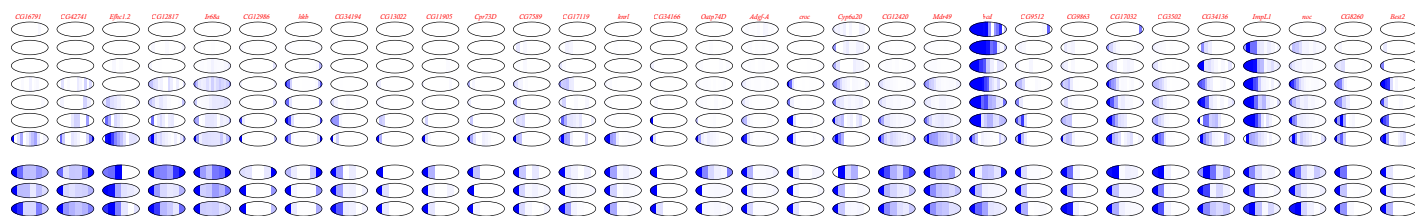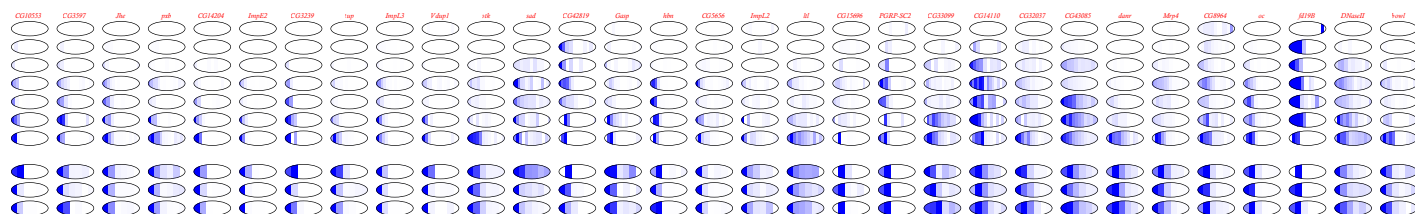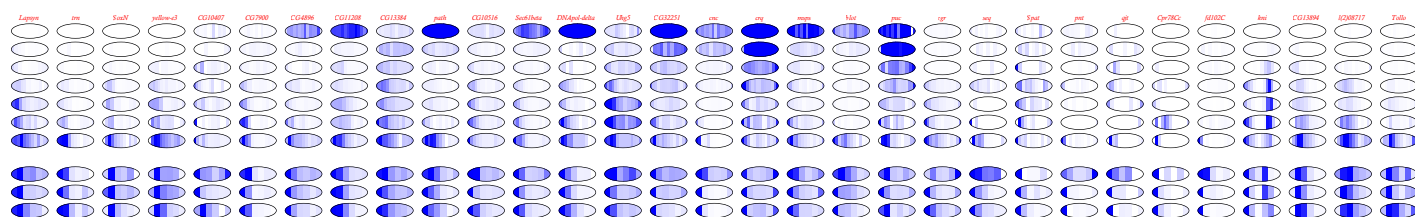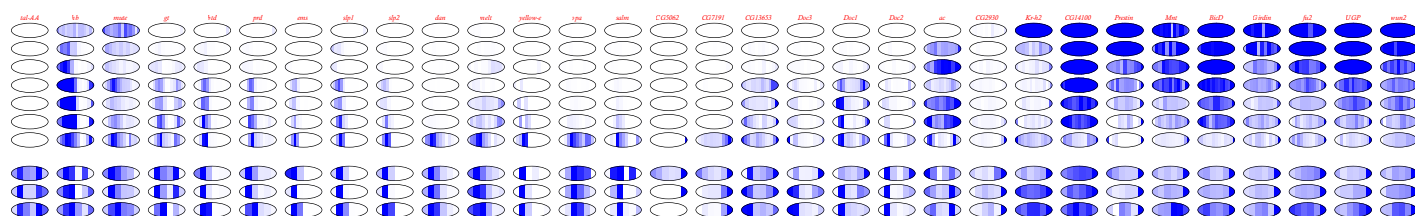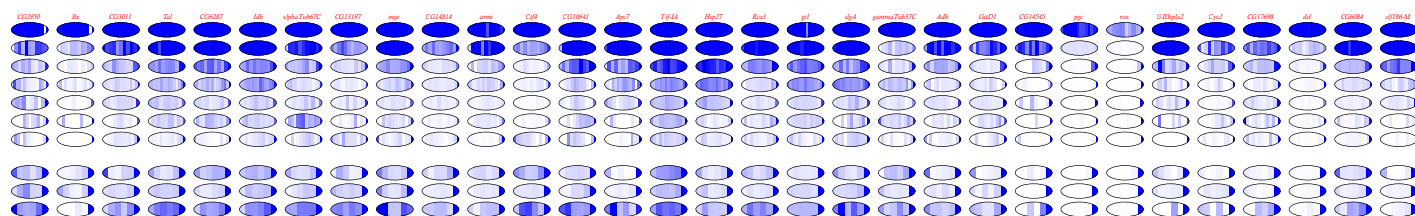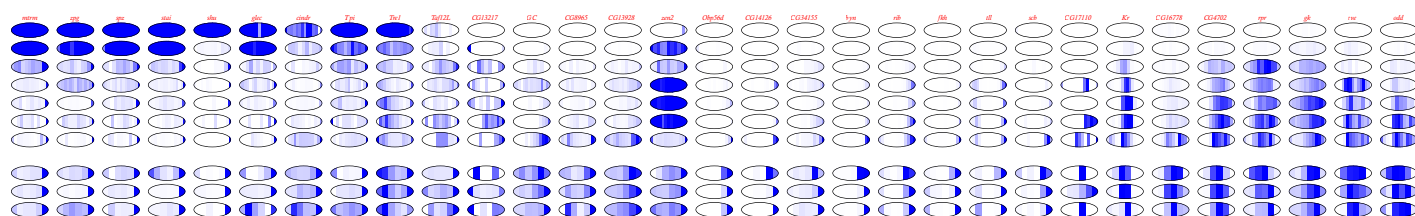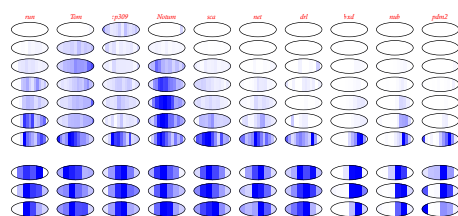

Supplement: Figure S7 — Data from 25 µm timecourse and 60 µm embryos for a large number of genes with manually curated patterns. (PDF) [file pone.0071820.s007.pdf]
